# Cancer‐Associated Fibroblasts Functions as Multifunctional Architects of the Tumor Stroma and Represent Emerging Therapeutic Vulnerabilities

**DOI:** 10.1002/advs.202510043

**Published:** 2026-03-28

**Authors:** Rujiao Liu, Lili Su, Shuiping Gao, Wenting Liu, Hongxia Wang

**Affiliations:** ^1^ Department of Medical Oncology Fudan University Shanghai Cancer Center Shanghai P. R. China; ^2^ Department of Oncology Shanghai Medical College Fudan University Shanghai P. R. China; ^3^ School of Health Science and Engineering University of Shanghai for Science and Technology Shanghai P. R. China

**Keywords:** CAFs, multifunctional, therapeutic challenges

## Abstract

Cancer‐associated fibroblasts (CAFs) are the predominant stromal components within the tumor microenvironment (TME), playing multifaceted roles in cancer progression through dynamic interactions with neoplastic and immune cells. Emerging evidence has revealed remarkable heterogeneity and plasticity of CAFs, which originate from diverse cellular precursors. This cellular diversity, coupled with dynamic epigenetic reprogramming and bidirectional cross‐talk with tumor cells, generates distinct CAF subsets with specialized functional outputs. Here, we systematically review the current understanding of CAF biology, encompassing their cellular origins, molecular heterogeneity, and the complex signaling networks. We discuss the functional of CAFs, detailing their protumorigenic roles in extracellular matrix (ECM) remodeling, immunosuppressive niche formation, metabolic reprogramming, angiogenesis, therapy resistance, and maintenance of cancer stem cell properties, while also highlighting emerging evidence for tumor‐restrictive CAF subsets. We critically evaluate therapeutic strategies targeting CAFs, including direct depletion approaches, ECM modulation, disruption of CAF‐tumor cross‐talk, and emphasis on clinical trials and associated challenges. Finally, we outline future directions leveraging single‐cell multiomics, patient‐derived models and combinatorial regimens to translate current understanding of CAF biology into effective stroma‐targeted therapies. This comprehensive framework not only positions CAFs as central architects of tumor ecosystems but also reveals actionable therapeutic vulnerabilities at the intersection of stromal biology and precision oncology.

## 1 Introduction

Cancer‐associated fibroblasts (CAFs), the principal stromal architects of tumor ecosystems, represent the most abundant and prominent cellular population within the tumor microenvironment (TME). These cells mediate pleiotropic effects on malignancy through dynamic reciprocity with neoplastic and immune compartments [1, 2]. As central mediators of TME reprogramming, CAFs orchestrate protumorigenic niches via paracrine growth factor signaling, extracellular matrix (ECM) remodeling, and conferral of therapy resistance [3, 4, 5, 6]. Emerging studies demonstrate that CAFs arise through tumor‐driven trans‐differentiation of diverse cellular precursors, including resident normal fibroblasts (NFs) [7], bone marrow‐derived mesenchymal stem cells (MSCs) [8], epithelial cells [9], stellate cells, adipocytes [10], and even endothelial cells [11]. As a common inhabitant of the TME, CAFs exhibit heterogeneous activation states marked by context‐dependent expression of phenotypic markers, including fibroblast activation protein (FAP), α‐smooth muscle actin (α‐SMA), platelet‐derived growth factor receptor (PDGFR), vimentin, podoplanin (PDPN), and fibroblast‐specific protein 1 (FSP1/S100A4) [1, 12, 13]. Notably, these markers display non‐overlapping expression patterns across CAF subpopulations, reflecting functional diversity. The absence of universal molecular identifiers hinders precise classification and functional mapping of CAF subtypes [14].

Despite recognition of the multifaceted effects of CAFs in oncogenesis, anti‐CAF strategies have faced significant challenges due to inherent heterogeneity of CAFs and context‐dependent roles that paradoxically exacerbate malignancy [15, 16, 17, 18]. CAFs exhibit dynamic phenotypic and functional plasticity, driven by bidirectional cross‐talk with neoplastic cells and other stromal components [1]. This functional adaptability is compounded by substantial intratumoral heterogeneity [19]. Recent advances in single‐cell technologies and spatial‐omics hold promise for addressing these challenges by decoding CAFs heterogeneity at unprecedented resolution. Investigations employing single‐cell RNA sequencing (scRNA‐seq) have delineated specialized CAF subsets characterized by unique transcriptional and functional specialization, including myofibroblastic CAFs (myCAFs; α‐SMA^+^POSTN^+^IL‐6^low^), inflammatory CAFs (iCAFs; PDGFRα^+^IL‐6^+^), antigen‐presenting CAFs (apCAFs; IFNγ^+^MHC‐II^+^), and vascular‐associated CAFs (vCAFs; CD146^+^SMA^+^), among others [19, 20, 21, 22, 23, 24, 25]. These functionally distinct subpopulations differentially regulate tumor progression and immune response through compartment specific mechanisms involving secretion of cytokines, chemokine, and ECM‐remodeling factors [20, 26]. CAF functional states also display spatiotemporal specificity. For instance, hypoxic niches selectively induce hypoxia‐sensitive senescent fibroblasts (hsCAFs) [27]. while STAT3^+^CXCL9^+^ CAFs localized within tertiary lymphoid structures (TLs) [28]. The cellular plasticity of CAFs is also reinforced by dynamic epigenetic remodeling [29]. The prevailing challenge in CAF‐targeted therapeutic development resides in resolving the functional hierarchy of biomarker heterogeneity and lineage‐committed CAF subsets within tumor ecosystems, while concurrently counteracting adaptive resistance mechanisms through compensatory signaling pathways.

In this review, we systematically summarize the pleiotropic functions of CAFs across cancer progression. We highlight the cellular heterogeneity of CAFs and their diverse functional capacities in driving tumor evolution. Furthermore, we critically evaluate CAFs as therapeutic targets and assess innovative strategies for stromal modulation in oncology. This integrative conceptual framework not only repositions CAFs as adaptive regulators of tumor ecosystems but also reveals actionable therapeutic vulnerabilities at the convergence of stromal biology and precision medicine.

## 2 Origins and Heterogeneity of CAFs

### 2.1 Cellular Lineage Diversification of CAFs

**FIGURE 1:**
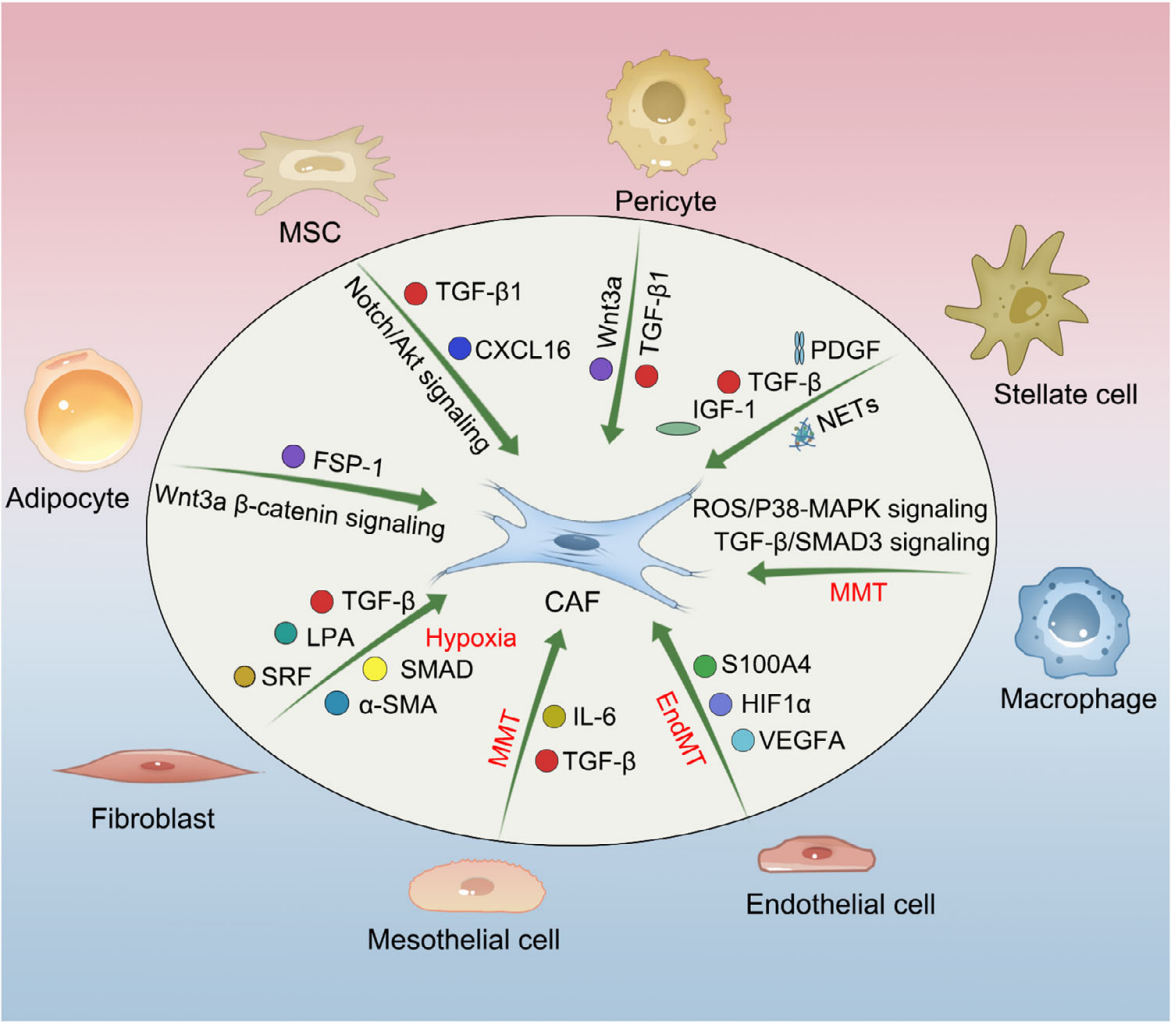
Cellular lineage diversification and molecular heterogeneity of cancer‐associated fibroblasts (CAFs). CAFs are not a homogeneous stromal entity but rather a heterogeneous collective of cells originating from distinct progenitor lineages via context‐dependent plasticity. Tissue‐resident fibroblasts are a primary source of CAFs; driven by tumor cell‐derived CM, hypoxic microenvironmental conditions, transforming growth factor beta (TGF‐β) family ligands, and lysophosphatidic acid (LPA), they undergo pathological reprogramming via autocrine‐paracrine signaling axes that amplify SMAD transcriptional activity and serum response factor signaling, characteristically orchestrating expression of the activation marker αSMA. Bone marrow‐derived mesenchymal stem cells (MSCs) are chemoattracted to the tumor niche by tumor‐ or stroma‐derived TGF‐β1 and CXCL16, differentiating into CAF subsets through the activation of Notch and Akt pathways. Mesothelial cells, regulated by the IL‐1/TGF‐β axis, undergo mesothelial‐to‐mesenchymal transition (MMT), losing mesothelial markers and transforming into antigen‐presenting cancer‐associated fibroblasts (apCAFs). At the invasive front of tumors, endothelial cells are induced by TGF‐β1, Wnt, Notch, and bone morphogenetic proteins (BMPs) to transform into CAFs via endothelial‐to‐mesenchymal transition (EndMT), accompanied by S100A4 overexpression—a process closely linked with vascular endothelial growth factor A (VEGFA) and hypoxia‐inducible factor 1‐alpha (HIF1α). Tumor‐derived Wnt3a drives adipocyte‐to‐fibroblast conversion via β‐catenin signaling, yielding adipocyte‐derived fibroblasts (ADFs) characterized by lipid metabolic reprogramming and high fibroblast‐specific protein 1 (FSP‐1) expression. Pancreatic stellate cells (PSCs) are activated by TGF‐β, PDGF, insulin‐like growth factor 1 (IGF‐1), and neutrophil extracellular traps (NETs) into α‐SMA+ CAFs, which secrete protumorigenic factors to enhance tumor survival and migration. Detaching from vascular structures promoted by TGF‐β1 and Wnt3a, pericytes transform into CAF effector phenotypes expressing fibronectin 1 (FN1), vimentin (VIM), and epithelial cell adhesion molecule (EPCAM), thereby increasing vascular permeability. Macrophages contribute to the CAF pool through macrophage‐to‐mesenchymal transition (MMT) mediated by TGF‐β/SMAD3 or reactive oxygen species (ROS) induced p38‐MAPK signaling.

CAFs are not a homogeneous stromal entity but rather a heterogeneous collective of cells originating from distinct progenitor lineages via context‐dependent plasticity. These activated stromal components are classically derived from resident NFs in native tissues, but they have also been documented to originate from trans‐differentiation of adipocytes, MSCs, pericytes, macrophages, stellate cells, epithelial cells, and mesothelial cells [30, 31, 32] (Figure 1). NFs‐derived CAFs demonstrate characteristic upregulation of POSTN (periostin) and COL1A1 (collagen type I alpha 1), whereas MSCs‐derived CAFs selectively express CD34 and PDGFRα [23, 33]. CAFs generated through epithelial‐mesenchymal transition (EMT) co‐express residual epithelial markers such as epithelial cell adhesion molecule (EpCAM) alongside acquired mesenchymal signatures including vimentin (VIM) and fibronectin 1 (FN1) [34]. Endothelial‐to‐CAF trans‐differentiation occurs preferentially at the invasive front of the tumors, which induced by transforming growth factor‐beta1 (TGF‐β1), suggesting that antiangiogenic agents may have a direct effect in decreasing activated fibroblasts [35, 36].

Resident Stromal Activation: Continuous observations of epithelial transformation sequences, from normal epithelia to atypical hyperplasia to invasive carcinoma, have demonstrated concurrent expansion and maladaptive activation of fibroblast populations. Tissue‐resident fibroblasts represent a primary source of CAFs, as evidenced by single‐cell profiling studies [7]. Experimental models revealed that cultured NFs transition into CAF‐like states under defined pathological stimuli, including exposure to tumor cell‐derived conditioned media or hypoxic microenvironmental conditions [37, 38]. This pathological reprogramming occurs through autocrine‐paracrine signaling axes, with key regulatory mediators including TGF‐β family ligands and lysophosphatidic acid [39, 40, 41, 42]. These effectors amplify SMAD (Sma‐ and Mad‐related protein) transcriptional activity and serum response factor signaling, collectively orchestrating expression of the activation marker αSMA [43].

MSC Recruitment: CAFs can originate from both local cellular reservoirs and bone marrow‐derived mesenchymal stem cells (BM‐MSCs) [8], as convincingly evidenced by integrated in vitro modeling and in vivo lineage‐tracing studies [11, 44]. Studies in murine tumor models demonstrate that transplanted BM‐MSCs undergo differentiation into functional CAFs [45]. In prostate cancer, tumor cell‐ and stroma‐derived TGF‐β1 mediates MSC recruitment to the tumor niche and induces their trans‐differentiation into CAF phenotypes [46]. Beyond TGF‐β1, other critical chemoattractants such as CCL2, CCL5, and CXCL12, along with tumor‐derived exosomes, have been implicated in directing MSC migration and subsequent CAF conversion [1]. Once incorporated, MSC‐derived CAFs contribute to tumor progression through paracrine signaling, including the secretion of CCL5, which can enhance cancer cell metastasis [8]. Notably, Lucia et al. identified a specific CAF subset co‐expressing FAP and ferroptosis suppressor protein‐1 (FSP‐1) that exhibits striking phenotypic and functional homology with bone marrow‐derived MSCs, suggesting that some CAF populations retain features of their mesenchymal progenitor origin [47].

EMT and Endothelial Trans‐differentiation: Epithelial cells and endothelial cells have been demonstrated to acquire fibroblastic phenotypes through EMT and endothelial‐to‐mesenchymal transition (EndMT), respectively, accompanied by S100A4 overexpression [9, 48, 49, 50]. During EndMT, endothelial cells lose their characteristic markers such as CD31 and VE‐cadherin while gaining mesenchymal markers such as FSP‐1 and α‐SMA, thereby contributing to the CAF pool [9]. Peritoneal mesothelial cells, a specialized epithelial population, undergo TGF‐β dependent trans‐differentiation into CAFs [51]. In pancreatic ductal adenocarcinoma (PDAC), mesothelial cells have been shown to transition specifically into apCAFs under the regulation of the IL‐1/TGF‐β axis, concurrent with the loss of mesothelial markers (e.g., MSLN) and the acquisition of fibroblastic signatures [32]. This transition highlights how specific cytokine milieus can direct distinct differentiation fates from the same progenitor population.

Adipocyte Conversion and Pericyte Shedding: Adipocytes, particularly white adipocytes [52, 53], constitute an alternative progenitor pool for CAFs, orchestrating lipid metabolic reprogramming in malignancies [53]. Tumor‐derived Wnt3a drives β‐catenin mediated adipocyte‐to‐fibroblast conversion, yielding adipocyte derived fibroblasts (ADFs) with concomitant FSP‐1 overexpression [10, 53]. These ADFs not only contribute to desmoplasia but also establish metabolic cross‐talk within the TME, influencing tumor cell behavior through altered lipid availability [53]. For pericytes, empirical support for CAF conversion remains limited [54, 55]. Tumor‐associated pericytes detach from vascular structures, adopting CAF‐like effector phenotypes that exacerbate vascular permeability [54]. This pericyte‐to‐CAF transition is often accompanied by the loss of pericyte markers (e.g., NG2, PDGFR‐β) and the gain of CAF‐associated markers such as α‐SMA, further diversifying the stromal landscape.

Emerging CAF Progenitor Populations: Recent studies leveraging lineage tracing and single‐cell technologies have elucidated the ontogeny of additional CAF subtypes, revealing an even broader cellular ancestry. In murine colitis‐associated colorectal cancer models, leptin receptor (Lepr)‐expressing pericryptal stromal precursors have been identified as a key progenitor population. These cells expand and differentiate into MCAM^+^ CAFs that foster an immunosuppressive tumor microenvironment, contributing to immune evasion and therapy resistance [7, 56, 57]. Separately, pancreatic stellate cells (PSCs), vitamin A‐storing cells resident in the pancreatic parenchyma, represent a major CAF progenitor in pancreatic cancer [8, 58]. Upon activation by tumor‐derived signals such as TGF‐β, PDGF, and IL‐1, PSCs undergo profound phenotypic conversion into α‐SMA^+^ CAFs [3, 8, 58]. High‐resolution single‐cell analyses have further revealed that PSCs contribute substantially to the myCAF pool in PDAC, establishing them as central players in the desmoplastic reaction characteristic of this malignancy [58]. The recognition of these diverse progenitor populations underscores the remarkable plasticity and context‐dependent nature of CAF differentiation.

### 2.2 Multidimensional Molecular Landscape of CAFs

The heterogeneity of CAFs has been systematically mapped across malignancies using advanced multiomics platforms, including scRNA‐seq, spatial transcriptomics, and proteomic profiling. These technologies have delineated specialized CAF subtypes with discrete molecular signatures, functional specializations, and pathway activations that collectively shape TME complexity (Table 1).

**TABLE 1:** Molecular subtypes and functional characteristics of cancer‐associated fibroblasts (CAFs).

CAF subtype	Key biomarkers	Cellular origin	Cancer type	Key signaling pathways	Primary functions	Clinical significance / Prognosis
**myCAF** (myofibroblastic CAF)	α‐SMA^high^, POSTN, TAGLN, THBS2, COL1A1/COL1A2, IL‐6^low^	Resident fibroblasts, stellate cells, MSCs	PDAC, BC, CRC, HCC	TGF‐β/SMAD2/3, MAPK, Rho‐GTPase	ECM remodeling, collagen deposition, biomechanical barrier formation, contractility, tumor invasion^58^	Generally associated with poor prognosis and desmoplasia; paradoxically, α‐SMA+ CAF depletion can accelerate PDAC progression^188^.
**iCAF** (inflammatory CAF)	PDGFRα^high^, IL‐6, CXCL12, LOX, CCL2, IL‐1	Resident fibroblasts, MSCs	PDAC, BC, HCC, NSCLC	JAK/STAT3, NF‐κB	Secretion of inflammatory cytokines, immune cell recruitment, modulation of immune microenvironment^60^.	Correlates with immunosuppression and therapy resistance; can be dynamically converted from myCAFs^60^.
**apCAF** (antigen‐presenting CAF)	HLA‐DR/DP/DQ, CD74, S100A4	Mesothelial cells, resident fibroblasts	PDAC, BC, CRC, HCC	IL‐1/TGF‐β, IFNγ	Antigen presentation to CD4+ T cells, induction of Treg differentiation, modulation of adaptive immunity 8, 25, 32, 61, 62, 63.	Dual role: may promote immune suppression via Treg induction; also associated with favorable prognosis in certain contexts 62.
**ifCAF** (interferon‐responsive CAF)	ISG15, MX1, IFIT1/2/3, STAT1, IRF7	Resident fibroblasts, MSCs	PDAC, BC, NSCLC	STING, JAK/STAT1, IRF	Tumor‐restraining properties, anti‐tumor immunity, neutrophil reprogramming 27, 98.	Associated with favorable prognosis; inducible by STING agonists^98^.
**CDCP1+FTL+ CAF**	CDCP1, FTL, FTH1, HIF1α	Resident fibroblasts	PDAC, HCC	HIF‐1α, ferroptosis pathway	Iron metabolism dysregulation, anti‐ferroptosis, metabolic adaptation to hypoxia 25.	Enriched in hypoxic niches; associated with poor prognosis and metabolic reprogramming
**ISG+ myCAF**	ISG15, MX1, α‐SMA, ACTA2	Resident fibroblasts	PDAC, BC	IFN signaling, TGF‐β	Interferon‐sensitive myofibroblastic phenotype, potential immunomodulatory functions 25.	Correlates with poor prognosis; represents hybrid myCAF/ifCAF state
**TSPAN8+ myCAF** (senescent CAF)	TSPAN8, α‐SMA, CDKN2A, p16^INK4a^	Resident fibroblasts	BC	p53/p21, senescence‐associated secretory phenotype (SASP)	Secretion of IL‐6/IL‐8, promotion of cancer stem cell properties, chemoresistance^65^.	Associated with therapy resistance and stemness maintenance.
**FAP+ CAF**	FAP, POSTN, COL1A1	Resident fibroblasts, MSCs	PDAC, BC, HCC, NSCLC	TGF‐β, Wnt/β‐catenin	ECM remodeling, immunosuppression, CD8+ T‐cell exclusion^1^, ^12^, ^13^, ^15^.	Negative prognostic marker; therapeutic target for FAP‐directed therapies.^202^, ^203^, ^204^, ^205^
**PDGFRα+ITGA11+ CAF**	PDGFRα, ITGA11, CHI3L1	Resident fibroblasts	BC	CHI3L1‐mediated signaling	Collagen deposition, vascular invasion, lymph node metastasis.	Promotes early vascular invasion; therapeutic target for CHI3L1/ITGA11 blockade^108^.
**MCAM+ CAF** (CD146+ CAF)	MCAM/CD146, α‐SMA, PDGFRβ	Pericryptal stromal precursors (Lepr+)	CRC, BC, GC	NF‐κB, CCL5	Context‐dependent: in BC maintains ER expression and tamoxifen sensitivity; in GC promotes M2 macrophage infiltration and immunosuppression.	Conflicting prognostic roles; protective in ER+ BC^179^, detrimental in GC^181^.
**SFRP2+ CAF**	SFRP2, COL1A1, FAP	Resident fibroblasts	CRC	Wnt antagonism, ECM remodeling	Treg differentiation, chemoresistance, matrix stiffening.	Associated with immunotherapy resistance and poor prognosis 72.
**LAM‐CAF** (lipid‐associated macrophage‐interacting CAF)	CXCL12, CXCR4, CCL5	Resident fibroblasts	TNBC, PDAC	CXCL12‐CXCR4, IL‐6/STAT3	Recruitment and polarization of lipid‐associated macrophages (LAMs), immunosuppression.	Enriched in ICI‐refractory TNBC; therapeutic target for LAM depletion^122^.
**VDR‐CAF** (vitamin D receptor‐expressing CAF)	VDR, α‐SMA, PDGFRβ	Stellate cells, resident fibroblasts	PDAC, GC	Vitamin D receptor signaling	Quiescent phenotype, reduced α‐SMA expression, decreased ECM production^215^, ^217^, ^218^.	Favorable when in quiescent state; VDR agonists (calcipotriol, paricalcitol) induce tumor‐suppressive phenotype.
**Meflin+ CAF** (restrictive CAF)	Meflin (ISLR), α‐SMA^low^	Pancreatic stellate cells, MSCs	PDAC	Vitamin D signaling, retinoic acid signaling	Tumor‐restrictive functions, maintenance of tissue homeostasis, improved collagen structure^175^, ^176^, ^178^.	Associated with favorable prognosis; represents quiescent or unactivated state.
**Cav‐1+ CAF** (Caveolin‐1+ CAF)	Caveolin‐1 (Cav‐1), α‐SMA	Resident fibroblasts	BC, ICC	TGF‐β/Smad, cyclin D1 antagonism	Dual role: in BC suppresses tumor migration and stemness^182^, ^183^; in ICC associated with poor prognosis and Treg infiltration^184^.	Context‐dependent prognostic significance; loss of Cav‐1 in BC CAFs promotes EMT^183^.
**rCAF** (restrictive CAF)	Meflin, CD146 (context‐dependent), Cav‐1 (context‐dependent)	Multiple origins	PDAC, BC	Homeostatic signaling pathways	Tumor suppression, maintenance of tissue homeostasis, “brake” function in early tumorigenesis^175^.	Generally associated with favorable prognosis; often outcompeted by pCAFs during progression.
**pCAF** (promoting CAF)	α‐SMA, FAP, PDGFRα, multiple subtype‐specific markers	Multiple origins	Pan‐cancer	TGF‐β, Wnt, Hedgehog, Notch, PDGF	Tumor promotion, immunosuppression, ECM remodeling, therapy resistance 175.	Dominant CAF phenotype in advanced tumors; associated with poor prognosis
**csCAF** (complement‐secreting CAF)	Complement factors (C3, C1s, CFD), S100A4	Resident fibroblasts	PDAC	Complement cascade	Complement secretion, potential tumor‐restraining properties 99.	Associated with favorable prognosis in PDAC.
**EndoCAF**	FAPα, CD144 (VE‐cadherin), CD31^low^	Endothelial cells (via EndMT)	Multiple cancers	TGF‐β1, EndMT	Vasculogenic mimicry, paracrine STAT3 signaling, metastasis^9^, ^35^, ^36^.	Enriched at invasive front; contributes to vascular abnormalities.
**ADF** (adipocyte‐derived fibroblast)	FSP‐1, Wnt3a, β‐catenin, PPARγ^low^	White adipocytes	BC, ovarian cancer	Wnt/β‐catenin, Wnt3a	Lipid metabolic reprogramming, desmoplasia, metabolic cross‐talk^10^, ^53^, ^54^.	Contributes to metabolic symbiosis and ECM remodeling.
**Lepr+ CAF**	Lepr (leptin receptor), MCAM	Pericryptal stromal precursors	CRC	Leptin signaling	Immunosuppressive TME formation, immune evasion, therapy resistance^7^, ^56^, ^57^.	Promotes immunosuppression in colitis‐associated CRC.
**PSC‐CAF** (pancreatic stellate cell‐derived CAF)	α‐SMA, desmin, GFAP, vimentin	Pancreatic stellate cells	PDAC	TGF‐β, PDGF, IL‐1, Hedgehog	Desmoplastic reaction, ECM production, tumor growth and metastasis^8^, ^58^.	Major contributor to PDAC stroma; therapeutic target for stellate cell reprogramming.

Abbreviations: Cancer types: BC, breast cancer; CRC, colorectal cancer; GC, gastric cancer; HCC, hepatocellular carcinoma; HNSCC, head and neck squamous cell carcinoma; ICC, intrahepatic cholangiocarcinoma; NSCLC, non‐small cell lung cancer; PDAC, pancreatic ductal adenocarcinoma; TNBC, triple‐negative breast cancer.

Key markers: α‐SMA, alpha‐smooth muscle actin; POSTN, periostin; TAGLN, transgelin; THBS2, thrombospondin 2; COL1A1/COL1A2, collagen type I alpha 1/2; PDGFRα/β, platelet‐derived growth factor receptor alpha/beta; IL‐6, interleukin‐6; CXCL12, C‐X‐C motif chemokine ligand 12; LOX, lysyl oxidase; CCL2, C‐C motif chemokine ligand 2; HLA‐DR/DP/DQ, human leukocyte antigen‐DR/DP/DQ; CD74, CD74 molecule; ISG15, ISG15 ubiquitin‐like modifier; MX1, MX dynamin‐like GTPase 1; IFIT1/2/3, interferon‐induced protein with tetratricopeptide repeats 1/2/3; STAT1/3, signal transducer and activator of transcription 1/3; IRF7, interferon regulatory factor 7; CDCP1, CUB domain‐containing protein 1; FTL/FTL1, ferritin light chain; FTH1, ferritin heavy chain 1; HIF1α, hypoxia‐inducible factor 1‐alpha; TSPAN8, tetraspanin 8; CDKN2A, cyclin‐dependent kinase inhibitor 2A; FAP, fibroblast activation protein; ITGA11, integrin subunit alpha 11; CHI3L1, chitinase 3‐like 1; MCAM/CD146, melanoma cell adhesion molecule; SFRP2, secreted frizzled‐related protein 2; VDR, vitamin D receptor; ISLR/Meflin, immunoglobulin superfamily containing leucine‐rich repeat; Cav‐1, caveolin‐1.

Pathways: ECM, extracellular matrix; EndMT, endothelial‐to‐mesenchymal transition; JAK/STAT, Janus kinase/signal transducer and activator of transcription; HIF‐1α, hypoxia‐inducible factor‐1 alpha; MAPK, mitogen‐activated protein kinase; NF‐κB, nuclear factor kappa‐B; PDGF, platelet‐derived growth factor; SASP, senescence‐associated secretory phenotype; SMAD, Sma‐ and Mad‐related protein; STING, stimulator of interferon genes; TGF‐β, transforming growth factor‐beta; Wnt, wingless‐type MMTV integration site.

Functions: TME, tumor microenvironment; Treg, regulatory T cell; EMT, epithelial‐mesenchymal transition; ICI, immune checkpoint inhibitor; LAM, lipid‐associated macrophage.

A foundational dichotomy exists between myCAFs and iCAFs, first characterized in PDAC and subsequently validated across multiple cancer types. myCAFs, defined by high α‐SMA expression and low IL‐6 production (α‐SMA^high^IL‐6^low^), localize proximal to cancer cells and demonstrate upregulated TGF‐β signaling targets such as CTGF and COL1A1 [58]. A distinctive feature of myCAFs is their hypercontractile phenotype and capacity to synthesize structural ECM components (e.g., collagens), forming biomechanical barriers that restrict tumor expansion and modulate therapeutic responses [59]. In contrast, iCAFs exhibit low α‐SMA expression with elevated inflammatory mediators including IL‐6, CXCL12, and PDGFRα, sustained JAK/STAT pathway activation, and distal positioning relative to tumor nests [58, 60]. This dichotomy reflects fundamental plasticity, with TGF‐β promoting myCAF differentiation and IL‐1α driving iCAF phenotypes [60]. In PDAC, Hedgehog pathway inhibition skews CAF heterogeneity toward iCAFs, fostering immunosuppression via cytotoxic T‐cell depletion and Treg expansion [61].

Beyond this core dichotomy, scRNA‐seq analyses have revealed a rare subset of MHC‐II^high^ (HLA‐DP/DQ/DR^+^) apCAFs with immunomodulatory potential [32, 62, 63, 64]. Originating from mesothelial cells via IL‐1/TGF‐β signaling in PDAC, apCAFs express canonical markers including CD74 [32, 63]. Tao et al. reported that apCAFs exhibit marked intra‐subtype heterogeneity, stratifying into five subsets co‐expressing apCAF markers and lineage‐specific immune genes, underscoring context‐dependent immune regulation [25]. Functionally, apCAFs can activate CD4^+^ T cells via MHC‐II presentation, yet in pancreatic tumors they may paradoxically expand regulatory T cells and dampen anti‐tumor immunity [32, 63].

Emerging evidence has identified functionally specialized CAF subsets across malignancies. Senescence‐associated TSPAN8^+^ myCAFs confer chemotherapy resistance by enhancing cancer cell stemness through IL‐6/IL‐8 secretion and metabolic reprogramming involving GLS1 and PYCR1 [65, 66]. Mechanistically, TSPAN8 promotes phosphorylation of ubiquitin E3 ligase RBBP6 via MAPK11 recruitment, leading to SIRT6 degradation and subsequent metabolic reprogramming that supports tumor outgrowth [65]. Lipid‐laden ABCA8a^+^ CAFs fuel tumor oxidative phosphorylation through lipid transfer in SETD2‐deficient pancreatic tumors [29]. In breast cancer, Cords et al. systematically classified nine CAF clusters, each encoding distinct molecular programs and functional outputs, with specific subsets associated with patient outcomes [67]. Costa et al. identified four CAF subsets (CAF‐S1 to CAF‐S4) in human breast cancer, with CAF‐S1 promoting immunosuppression by attracting CD4^+^CD25^+^ T cells and enhancing regulatory T‐cell differentiation [68]. In non‐small cell lung cancer, Lambrechts et al. identified five CAF subsets via scRNA‐seq, including clusters enriched in tumors versus non‐malignant tissues with distinct myogenesis and angiogenesis activities [69].

Spatial analysis has revealed conserved organizational principles governing CAF distribution. Breast cancer studies delineated four conserved CAF subtypes with unique spatial distributions [28]. In hepatocellular carcinoma, proteomic mapping distinguished FAP^+^POSTN^+^ CAF‐FAPs enriched in intratumoral inflammatory niches, promoting CD8^+^ T‐cell exhaustion via PD‐1/PD‐L1 interactions, from C7^+^PDGFRA^+^ CAF‐C7s localized to peri‐tumoral repair microdomains that drive macrophage SPP1‐mediated immunosuppression [70]. Additional specific CAF subtypes with compartmentalized functions have been characterized across malignancies, including RGS5^+^ myCAFs associated with liver metastasis and poor prognosis in PDAC [71], and SFRP2^+^ CAFs that induce Treg differentiation and ECM remodeling in colorectal cancer, linked to chemoresistance. These advances not only decode CAFs heterogeneity but also establish actionable frameworks for developing stroma‐targeted therapeutic strategies to disrupt protumorigenic networks and potentiate treatment efficacy [71, 72].

## 3 Cancer Cell‐CAFs Cross‐Talk via Multimodal Activation Pathways

**FIGURE 2:**
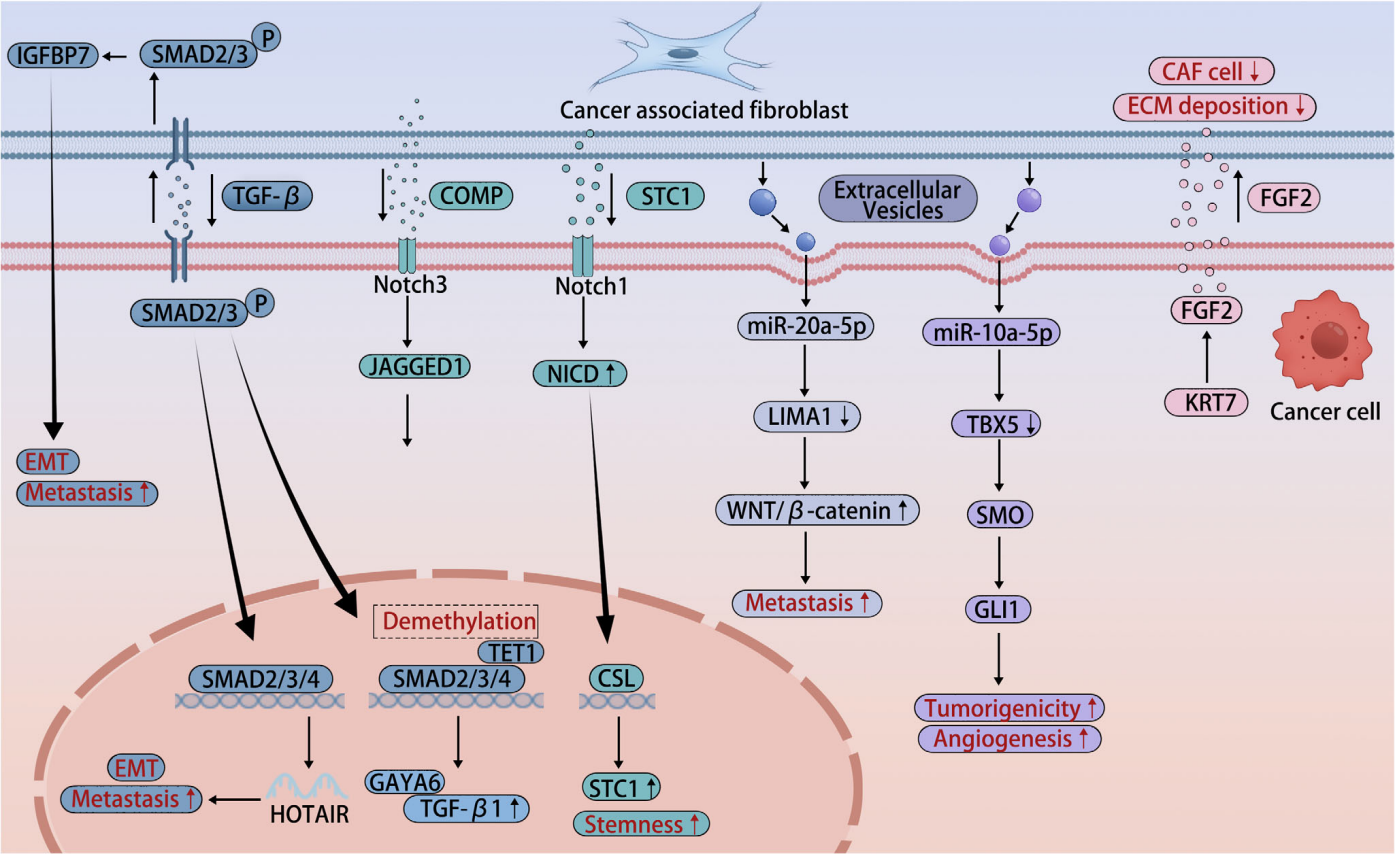
Molecular mechanisms of cancer cell‐CAF (cancer‐associated fibroblast) cross‐talk via multimodal activation pathways. Tumor‐secreted transforming growth factor beta 1 (TGF‐β1) activates CAFs to overexpress and secrete insulin‐like growth factor binding protein 7 (IGFBP7) via SMAD2/3 signaling, which in turn enhances tumor cell epithelial‐mesenchymal transition (EMT), stemness, and metastatic potential. The SMAD2/3/4 complex transcriptionally activates the lncRNA HOTAIR, accelerating metastatic progression. Concurrently, TET1‐mediated demethylation of the SMAD4 promoter enhances SMAD4 expression, establishing a GATA6‐dependent feedforward loop that sustains TGF‐β upregulation. In the Notch signaling cascade, cartilage oligomeric matrix protein (COMP) activates Notch3 to induce JAGGED1 and EMT, while stanniocalcin 1 (STC1) triggers Notch1, leading to Notch intracellular domain (NICD) nuclear translocation and binding with CSL to maintain cancer cell stemness. Extracellular vesicles (EVs) facilitate intercellular communication: exosomal miR‐20a‐5p downregulates LIM domain and actin‐binding 1 (LIMA1), resulting in aberrant Wnt/β‐catenin activation and metastasis; exosomal miR‐10a‐5p targets TBX5 to relieve inhibition on smoothened (SMO), activating GLI family zinc finger 1 (GLI1) mediated Hedgehog signaling to enhance tumorigenicity and angiogenesis. Additionally, keratin 7 (KRT7) promotes fibroblast growth factor 2 (FGF2) expression, which acts to suppress CAF proliferation and reduce extracellular matrix (ECM) deposition.

The reciprocal interplay between cancer cells and CAFs establishes a self‐reinforcing signaling ecosystem that drives malignant progression through evolutionarily conserved pathways. including the TGF‐β/SMAD, Wnt/β‐catenin, Hedgehog (Hh), Notch, and PDGF/PDGFR pathways, which collectively orchestrate the bidirectional cross‐talk that sustains CAF activation and promotes tumor growth, metastasis, and therapy resistance (Figure 2).

TGF‐β/SMAD Axis: The canonical TGF‐β/SMAD axis drives ECM remodeling through transcriptional upregulation of type I collagen and α‐SMA [73]. In PDAC, tumor‐secreted TGF‐β1 activates CAFs via phosphorylated SMAD2/3 signaling, concurrently promoting MAPK pathway activation [74]. TGF‐β1 from cancer cells stimulates myCAFs to upregulate IGFBP7 through SMAD2/3 phosphorylation, thereby potentiating EMT and metastatic dissemination [75]. Conversely, CAF‐derived TGF‐β1 transcriptionally activates the lncRNA HOTAIR via SMAD2/3/4 complex, accelerating EMT and BC metastasis [76]. TET1‐mediated demethylation of the SMAD4 promoter transcriptionally activates GATA6, establishing a self‐amplifying loop that sustains TGF‐β signaling and CAF activation [77]. Furthermore, CAFs coordinate SMAD4‐dependent oncogenesis via the TGFβ1‐LAMC1‐SP1 axis, driving tumor proliferation, ECM remodeling, and iCAF differentiation through NF‐κB‐CXCL1‐STAT3 circuitry [78]. These findings position the TGF‐β/SMAD axis as both a central regulator of CAF‐driven tumorigenesis and a tractable therapeutic node, though clinical translation has been challenged by the pathway's context‐dependent duality, acting as a tumor suppressor in early stages while promoting progression in advanced disease.

Wnt/β‐Catenin Feedforward Loop: Dysregulated Wnt/β‐catenin signaling coordinates malignant progression across cancer types, governing initiation, development, and metastatic dissemination [79]. CAFs exploit Wnt/β‐catenin cross‐activation with TGF‐β and Notch pathways to sustain EMT and therapeutic recalcitrance. CAF‐secreted DKK1, a Wnt antagonist, suppresses NK cell cytotoxicity via β‐catenin dependent inhibition of AKT/ERK/S6 phosphorylation, facilitating BC immune evasion [80]. CAF‐derived exosomal miR‐20a‐5p targets LIMA1 to activate Wnt signaling and accelerate HCC metastasis [81]. Environmental xenobiotics (e.g., BPA/BPS) activate Wnt/β‐catenin in ovarian cancer cells, inducing SPP1‐driven CAF transformation and ECM restructuring [82]. The Wnt pathway also regulates CAF heterogeneity, with β‐catenin signaling promoting myofibroblastic differentiation while its inhibition skews toward inflammatory phenotypes [72].

Hedgehog (Hh) Stromal Reprogramming: CAFs mediate Hh‐driven stromal reprogramming through paracrine SHH ligand secretion and nuclear GLI‐1 activation, amplifying tumor‐stroma symbiosis [83]. In PDAC, Hh pathway activation in myCAFs stimulates tumor growth, whereas its inhibition skews CAF heterogeneity toward iCAFs, fostering immunosuppression via cytotoxic T‐cell depletion and Treg expansion [61]. This context‐dependent duality highlights the complexity of Hh signaling in the TME, where pathway inhibition can paradoxically accelerate progression by altering CAF subset composition [84]. Cervical cancer CAFs release EVs containing miR‐10a‐5p, which activates Hh signaling through TBX5 suppression to drive angiogenesis and tumorigenesis [61]. Gastric cancer progression correlates with Hh pathway activation, aggressive phenotypes, and immune evasion [85]. These findings underscore Hedgehog signaling as a central axis in CAF‐driven tumor ecosystems, with therapeutic implications that require careful consideration of subset‐specific effects [86]

Notch Pathway: Canonical Notch signaling initiates through ligand‐induced receptor cleavage, enabling nuclear translocation of the Notch intracellular domain (NICD) that displaces CSL corepressors to activate target genes transcription [87]. CAF‐derived STC1 activates the Notch signaling pathway by directly binding to the Notch1 receptor, inducing the nuclear translocation of the NICD and its subsequent interaction with the transcription factor CSL (RBP‐J). This process directly initiates STC1 transcription within hepatocellular carcinoma cells, thereby establishing a stromal‐tumor amplifying feedforward signaling loop that sustains tumor stemness [88]. CAFs activate Notch signaling via ligand secretion to drive tumor invasion and TME reprogramming. In ovarian cancer, CAF‐derived COMP potentiates Notch3 signaling, promoting EMT and metastatic spread [89]. CAFs secrete IL‐6 to induce Notch via JAK2/STAT3/c‐MYC axis, driving neuroendocrine‐to‐non‐neuroendocrine transition and chemoresistance in small cell lung cancer. Lian et al. identified CEMIP^+^NKD1^+^ CAFs that stimulate melanocyte proliferation via JAG1‐NOTCH1/3 interactions, exacerbating EMT and immunotherapy resistance [90]. Paradoxically, apoptotic cancer cells reprogram CAFs via Notch1‐WISP‐1 signaling to suppress metastasis, revealing context‐dependent duality [91]. This bidirectional regulation underscores the nuanced role of Notch signaling in CAF biology, where pathway activation can yield opposing outcomes depending on cellular context and ligand‐receptor pairing [4, 72].

PDGF/PDGFR Axis: The PDGF/PDGFR axis coordinates stromal dynamics through kinase‐mediated cascades. PDGF receptors localize to perivascular cells, fibroblasts, and myofibroblasts within tumor stroma. Beyond direct fibroblast stimulation, PDGF signaling recruits pericytes and myofibroblasts to remodel stromal architecture, fostering protumorigenic niches [92]. PDGF‐BB binding to PDGFR‐β induces fibroblast activation via IKK/NF‐κB signaling, upregulating α‐SMA and FAP‐α to promote oral squamous cell carcinoma invasion [93]. In PDAC, PDGF‐activated CAFs exhibit attenuated ECM deposition via FGF2 secretion, facilitating metastatic niche formation [94]. Cervical cancer‐derived PDGF activates PDGFR‐expressing CAFs to upregulate fibroblast growth factor, driving angiogenesis and tumor proliferation [95].

## 4 The Protumorigenic and Antitumorigenic Functions of CAFs

**FIGURE 3:**
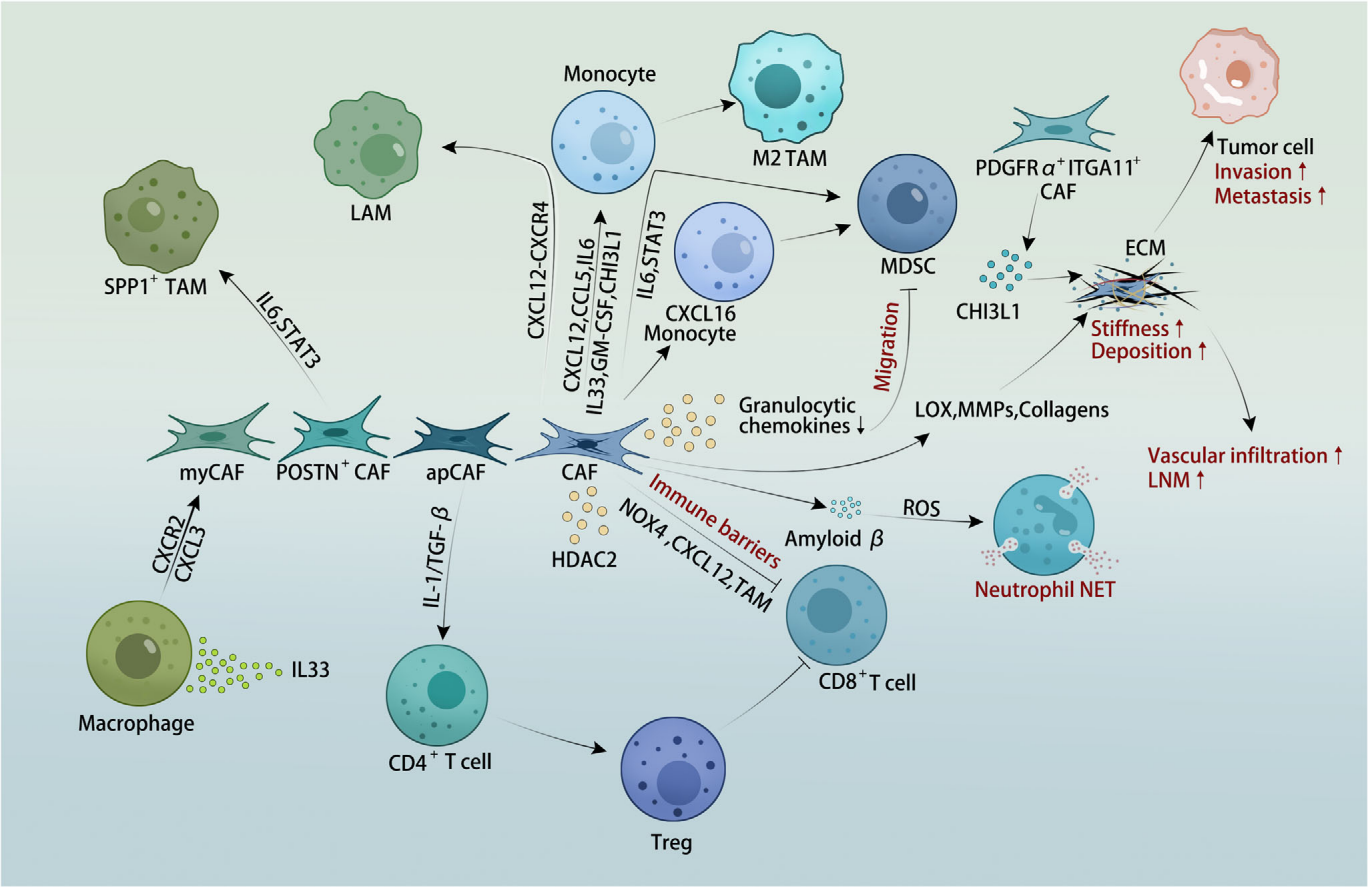
The protumorigenic functions of **cancer‐associated fibroblasts** (CAFs): Immunosuppressive niche formation and extracellular matrix (ECM) remodeling. CAFs orchestrate a multifaceted immunosuppressive microenvironment and restructure the physical matrix to drive tumor progression. Immunosuppressive niche formation: IL‐33‐stimulated macrophages secrete CXCL3 to induce myCAF activation via CXCR2. Reciprocally, POSTN^+^ CAFs drive SPP1^+^ tumor‐associated macrophage (TAM) polarization through IL‐6/STAT3, while the CXCL12‐CXCR4 axis promotes the differentiation of monocytes into lipid‐associated macrophages (LAMs). CAFs further recruit and polarize monocytes into M2‐like TAMs via CXCL12, CCL5, IL‐6, IL‐33, GM‐CSF, and CHI3L1. Antigen‐presenting cancer‐associated fibroblasts (apCAFs) induce regulatory T cell (Treg) generation from CD4^+^ T cells via IL‐1/TGF‐β signaling. Distinct mechanisms suppress CD8^+^ T cells: CAFs establish immune barriers that inhibit CD8^+^ T‐cell infiltration, thereby promoting resistance to immune checkpoint inhibitors (ICIs). This suppression is mediated directly via NADPH oxidase 4 (NOX4) and CXCL12, and synergistically through interactions with TAMs to drive exhaustion. Regarding myeloid‐derived suppressor cell (MDSC) expansion, distinct pathways are involved: (1) CAFs recruit CXCL16‐responsive monocytes which differentiate into MDSCs; (2) the IL‐6/STAT3 axis promotes monocyte‐to‐MDSC differentiation; and (3) histone deacetylase 2 (HDAC2) regulates this process by downregulating granulocytic chemokines, which limits the migration of MDSCs. Additionally, CAF‐secreted amyloid‐β triggers reactive oxygen species (ROS) dependent neutrophil extracellular trap (NET) formation. ECM remodeling: PDGFRα^+^ITGA11^+^CAFs secrete CHI3L1 to enhance ECM stiffness and collagen deposition, promoting vascular infiltration and lymph node metastasis (LNM). Concurrently, CAF‐mediated secretion of lysyl oxidase (LOX), metalloproteinases (MMPs), and collagens restructures the matrix to drive tumor invasion and metastasis.

The diversity of precursor cells and the varied cytokines, chemokines, and growth factors secreted into the TME contribute to CAF heterogeneity, which can exert either tumor‐promoting or tumor‐restrictive effects [72, 96]. Tumor tissues exhibit significantly higher stiffness than normal tissues, a biomechanical property of the ECM that drives malignant progression by limiting drug penetration, impeding immune cell infiltration, and activating integrin‐mediated focal adhesion kinase (FAK/SRC) signaling and mechanotransduction pathways (e.g., ERK, Rho‐GTPase, YAP/TAZ) [97]. CAF‐driven functions span multiple dimensions of the TME (Figure 3), with the majority of evidence supporting protumorigenic roles while emerging studies highlight the existence of functionally distinct subsets with tumor‐restraining properties [98, 99].

### 4.1 Protumorigenic Functions of CAFs

#### 4.1.1 ECM Remodeling and Biomechanical Stress

CAFs increase ECM matrix stiffness by secreting structural components (e.g., collagens, fibronectin) and simultaneously degrade its normal architecture through production of remodeling enzymes such as lysyl oxidase (LOX) and matrix metalloproteinases (MMPs), collectively reshaping a desmoplastic stroma [100, 101, 102]. In papillary thyroid carcinoma, CAF‐mediated collagen deposition increases peritumoral stiffness, correlating with lymph node metastasis and poor prognosis [103]. Among 107 colorectal cancer patients, the median overall survival of the low‐stiffness group significantly exceeded that of the high‐stiffness group [104]. CAFs facilitate cancer cell invasion through protease‐dependent and force‐mediated ECM reorganization [105]. This remodeling not only provides physical guidance for cancer cell migration but also activates proinvasive signaling pathways through EV‐mediated transfer of oncogenic cargo, ultimately establishing pre‐metastatic niches in distant organs [106, 107]. Zheng et al. identified PDGFRα^+^ITGA11^+^ CAFs that promote vascular invasion and lymph node metastasis via CHI3L1‐mediated collagen deposition; combinatorial CHI3L1/ITGA11‐neutralizing antibodies inhibit early breast cancer vascular invasion [108]. Hyaluronic acid (HA) interacts with CD44 to upregulate drug efflux transporters, inducing chemotherapy resistance, a phenotype reversible by transferring organoids to low‐stiffness matrices [109]. Caveolin‐1 (Cav‐1), a membrane‐associated scaffold protein, critically regulates this process; Cav‐1‐deficient mice exhibit stromal disorganization [110].

#### 4.1.2 Immunosuppressive Niche Formation

CAF‐shaped immunosuppressive TMEs drive resistance to immune checkpoint inhibitors (ICIs) across cancers, interacting with multiple immune cell types, including effector T cells, regulatory T cells (Tregs), tumor‐associated macrophages (TAMs), myeloid‐derived suppressor cells (MDSCs), and tumor‐associated neutrophils (TANs) [111].

Regarding effector T cells and Tregs, CAFs establish physical and chemical immune barriers that suppress CD8^+^ T‐cell infiltration into tumors, thereby promoting resistance to ICIs [112, 113]. CAF abundance inversely correlates with CD8^+^ tumor‐infiltrating lymphocytes (TILs) [114]. Immunosuppressive mechanisms include secretion of IL‐6, CXCL9, CXCL12, and TGF‐β, as well as PD‐L1‐mediated T‐cell inhibition [37, 115]. In PDAC, FAPα^+^ CAF‐derived CXCL12 is a principal immunosuppressive driver [116, 117]. Antigen‐presenting CAFs (apCAFs) polarize naïve CD4^+^ T cells into Tregs via IL‐1/TGF‐β signaling, thereby suppressing CD8^+^ T‐cell function [32]. NOX4 inhibition or targeting the IGF2/IGF1R signaling pathway with the inhibitor linsitinib can significantly reverse CAF‐mediated CD8^+^ T‐cell exclusion, enhancing ICI efficacy [118, 119].

With respect to myeloid cells, CAFs establish premetastatic niches (PMNs) via EV‐mediated tissue remodeling [106] and recruit monocytes, polarizing them into M2‐like TAMs through CXCL12, CCL5, IL‐6, IL‐33, GM‐CSF, and CHI3L1 [120, 121]. In triple‐negative breast cancer, CAFs reprogram monocytes into immunosuppressive lipid‐associated macrophages (LAMs) via CXCL12‐CXCR4 signaling, a population expanded in ICI‐refractory patients; LAM depletion suppresses tumor growth in preclinical model [122]. POSTN^+^ CAFs induce SPP1^+^ TAM polarization through IL‐6/STAT3 signaling [123]. TAMs reciprocally modulate CAFs: in PDAC, IL‐33‐activated macrophages secrete CXCL3 to drive myCAF transition via CXCR2 [124]. CAF‐TAM synergy drives CD8^+^ T‐cell exhaustion in hepatocellular carcinoma [125]. CAFs also promote monocyte‐to‐MDSC differentiation via IL‐6/STAT3 signaling [126, 127] and recruit CXCL16‐responsive monocytes that differentiate into monocytic MDSCs, exacerbating triple‐negative breast cancer aggressiveness [128]. HDAC2 downregulates CAF‐derived granulocytic chemokines, limiting PMN‐MDSC migration [129]. CAF‐secreted amyloid β induces neutrophil extracellular trap (NET) formation via reactive oxygen species (ROS) mediated pathways [130], while CLCF1 stimulates tumor cell‐derived CXCL6/TGF‐β to recruit and polarize N2 TANs [131].

#### 4.1.3 Metabolic Symbiosis and Nutrient Reprogramming

Emerging evidence demonstrates that CAFs act as metabolic master regulators, orchestrating tumor cell metabolic reprogramming through bidirectional cross‐talk mediated by exosomal metabolite shuttling and paracrine signaling pathways [29, 132]. In SETD2‐deficient pancreatic tumors, ABCA8a^+^ lipid‐rich CAFs transfer lipids to sustain tumor oxidative phosphorylation [29]. CAF‐derived acetate activates the ACSS2‐SP1‐SAT1 axis via histone acetylation, fueling PDAC survival and proliferation [133]. FSP+ CAFs enhance tumor glycolysis via FAK signaling; FAK‐depleted CAFs upregulate CCL6, CCL11, CCL12, and pentraxin‐3, suppressing glycolysis [125, 132]. CAF exosomal LINC01711 and HGF drive glycolysis in breast cancer and head and neck squamous cell carcinoma, respectively, elevating extracellular lactate [134, 135]. Spatial transcriptomics links CAF‐tumor cross‐talk to glycolytic and EMT pathway activation at PDAC invasive fronts [136]. CAF‐secreted WNT5A upregulates HK2 to enhance gastric cancer glycolysis [137]. Targeting PKMζ in hepatic stellate cells or Src SH3‐mediated Glut1 trafficking suppresses CAF activation and metastasis [138, 139].

Regarding glutamine metabolism, Gln‐deprived CAFs activate macropinocytosis to scavenge extracellular proteins, releasing amino acids for pancreatic cancer survival [140]. CAF exosomal METTL3 stabilizes SLC7A5 via m6A modification, enhancing NSCLC glutaminolysis [141]. Stromal PYCR1 converts glutamine to proline for collagen deposition [142], while NUFIP1‐driven autophagy enables PDAC proliferation via nucleoside secretion [143]. These pathways collectively highlight CAFs as metabolic hubs that sustain tumor fitness and therapy resistance.

Importantly, CAF‐derived EVs serve as critical mediators of metabolic cross‐talk [144]. By secreting EVs, CAFs deliver functional cargo, including proteins such as ATP6V1C1 [145], circular RNAs such as circTAX1BP1 [146], and various miRNAs [147], to tumor cells, directly activating key intracellular signaling pathways (e.g., TGF‐β/SMAD, IGF1R/Akt/ERK). These signals induce EMT, enhance invasive capacity, promote metabolic reprogramming, and lead to therapy resistance [148, 149]. Notably, CAF‐EVs construct a self‐reinforcing, prometastatic positive feedback loop: for instance, CAF‐EV‐delivered circTAX1BP1 upregulates TGF‐β secretion from tumor cells, which in turn activates specific CAF subpopulations (e.g., ITGA11^+^ myofibroblasts), prompting them to secrete more EVs carrying prometastatic molecules [150]. Similarly, proteins such as TSG6 on the surface of CAF‐EVs amplify TGF‐β signaling, further activating CAFs and suppressing CD8^+^ T‐cell function [149]. This bidirectional feedback loop continuously reinforces itself, persistently remodeling the local microenvironment and even creating conducive pre‐metastatic niches in distant organs [148, 150].

#### 4.1.4 Angiogenesis and Vascular Mimicry

CAFs drive angiogenesis by secreting a repertoire of angiogenic regulators including VEGFA, PDGF‐C, FGF2, CXCL12, and WNT2 [151, 152]. Pharmacological inhibition of PDGFR signaling using imatinib effectively suppresses invasive carcinoma progression [153]. FHL2‐expressing CAFs facilitate endothelial tube formation and metastatic dissemination through osteopontin upregulation, concurrently enhancing cancer cell motility and angiogenic activation [154]. WNT2 overexpression significantly elevates tumor vascular density and volume, inducing transcriptional upregulation of proangiogenic mediators including angiopoietin‐2, IL‐6, G‐CSF, and placental growth factor [155]. Orimo et al. demonstrated that CAF‐derived CXCL12 mediates endothelial progenitor cell recruitment into tumors, a process reversible upon CXCL12 neutralization [156]. In HCC, VEGFA^+^ CAFs directly stimulate intratumoral angiogenesis via paracrine interactions with capillary endothelial cells [157]. The CXCR4/CXCL12 axis in TNBC CAFs coordinately promotes angiogenic niche formation and chemoresistance, underscoring its dual role in metastatic progression [158].

#### 4.1.5 Therapy Resistance

CAFs confer resistance to chemotherapy, radiotherapy, and immunotherapy through multiple mechanisms spanning metabolic rewiring, exosome‐mediated signaling cascades, and dynamic stromal cross‐talk [159, 160]. The stiffened ECM creates a physical barrier that impedes drug delivery by compressing surrounding vasculature [159, 161].

Regarding chemoresistance, NF‐κB activation via GPR77‐mediated complement signaling induces a CD10+GPR77^+^ CAF subset that promotes chemoresistance by sustaining cancer stem cells through IL‐6 and IL‐8 secretion [160]. Stromal inflammation driven by CAF‐neutrophil interactions, such as those mediated through the CXCL1‐TNFα axis, perpetuates immunosuppressive niches [162]. Elevated IL‐6 levels in cancer cell‐CAF co‐cultures correlate with treatment resistance and disease progression [114, 163]. Nuclear receptor activation in CAFs associates with chemotherapy resistance, while combinatorial therapy with NR antagonists LE135 and bicalutamide significantly improves therapeutic efficacy [164]. Under high‐dose chemotherapy, CAFs secrete chemokines mediating treatment resistance, whereas low‐dose metronomic therapy suppresses these protumorigenic signals [165]. CAF‐secreted SPP1 and COL8A1 activate bypass signaling (RAF/MAPK, PI3K/AKT) and EMT, conferring tyrosine kinase inhibitor resistance in HCC and CRC [166, 167]. Patients with PDPN^+^ CAFs demonstrate significantly reduced EGFR‐TKI response rates [168].

For radioresistance, CAFs promote cancer cell survival through IL‐6/STAT3 axis activation, while supporting breast cancer progression via HGF and FGF2 secretion [169]. CAF‐derived IL‐6 activates STAT3 signaling, enhancing breast cancer cell survival post‐irradiation [169]. Similarly, CAF‐secreted HGF induces c‐Met pathway activation, driving EMT and radioresistance [170]. In CRC, CAF‐derived exosomal microRNA‐590‐3p upregulates PI3K/Akt signaling, conferring radioresistance [171]. CAF‐secreted CXCL1 enhances ROS accumulation and DNA damage response activation, fostering radioresistance [171]

#### 4.1.6 Maintenance of Cancer Stem Cell Properties

CAFs maintain cancer stemness through multifaceted mechanisms including paracrine signaling and exosomal cargo transfer, thereby driving chemotherapeutic resistance and tumorigenic potential. CAF‐derived miR‐146a‐5p suppresses ARID1A and PRKAA2 expression, activating STAT3/mTOR signaling cascades to enhance stemness properties and chemotherapy resistance in bladder cancer [66]. Fan et al. reported that senescent TSPAN8^+^ myCAFs secrete IL‐6 and IL‐8 to amplify cancer stem cell characteristics, promoting chemoresistance in breast cancer [65]. Conditioned medium derived from CAFs enhances spheroid colony formation and upregulates CSC marker expression; these pro‐stemness effects are attenuated through TGF‐β pathway inhibition [172]. Loss of exosomal miR‐34c‐5p in CAFs sustains stem‐like phenotypes in laryngeal carcinoma [173]. In triple negative breast cancer murine models, neoplastic cells secrete Hedgehog ligands that epigenetically reprogram CAFs to foster chemotherapy‐resistant CSC phenotypes through FGF5 overexpression and elevated fibrillar collagen deposition [174].

### 4.2 Antitumorigenic Functions of CAFs

While the majority of research demonstrates protumorigenic roles for CAFs, emerging evidence indicates that specific CAF subpopulations can exert tumor‐restrictive effects under certain microenvironmental contexts [98, 99]. These restrictive CAFs (rCAFs) play a crucial “brake” role in disease equilibrium, though their functions are often outcompeted by promoting CAFs (pCAFs) during tumor progression [175, 176]. Studies have shown that α‐SMA‐expressing CAFs can exert tumor‐suppressive effects in pancreatic cancer models, with depletion of α‐SMA^+^ CAFs paradoxically leading to more aggressive tumor phenotypes [177]. Profiling the heterogeneity of PDAC FAP^+^ stromal cells identified a previously undescribed interferon‐responsive CAF (ifCAF or irCAF) subtype with tumor‐restraining properties [98]. STING agonists (e.g., DMXAA/MSA‐2) promote the formation of this irCAF phenotype both in vitro and in vivo, suggesting potential therapeutic strategies to induce protective CAF states. Another PDAC study revealed tumor‐restraining complement‐secreting CAFs (csCAFs) associated with favorable prognosis [99].

Several markers have been associated with rCAF phenotypes. Meflin (ISLR), a glycosylphosphatidylinositol‐anchored cell surface protein, is a relatively well‐defined marker of quiescent or unactivated fibroblasts associated with benign tissue architecture [178]. Meflin‐positive CAFs appear around neoplastic cells even in early stages of PDAC (e.g., acinar‐to‐ductal metaplasia), acting as “sentinels” with inhibitory functions [176]. Meflin^+^ CAF infiltration significantly correlates with better patient prognosis, potentially by reducing α‐SMA^+^ CAF infiltration and improving collagen structure within the TME. Meflin expression is regulated by vitamin D, suggesting that rCAFs may have unique functional states amenable to therapeutic modulation.

CD146 (MCAM) demonstrates context‐dependent functions: in breast cancer, CD146^+^ CAFs maintain tumor cell ER expression, preserving estrogen dependence and tamoxifen sensitivity while correlating with better patient prognosis [179]. Loss of CD146 activates NF‐κB signaling, leading CAFs to secrete more protumorigenic factors such as CCL5, enhancing cancer cell migration and invasion [180]. However, in gastric cancer, single‐cell RNA sequencing identified a CD146^+^ CAF subpopulation closely associated with poor prognosis and M2‐like macrophage infiltration, suppressing CD8^+^ T‐cell function [181]. Thus, CD146 is not a universal protective marker and requires context‐specific interpretation.

Caveolin‐1 (Cav‐1) acts as a breast tumor suppressor by regulating epithelial cell proliferation and stromal microenvironment homeostasis, exerting dual protective effects against hyperplasia and tumorigenesis in both mammary epithelial and stromal cells through cyclin D1 antagonism [182]. Downregulation of Cav‐1 in CAFs promotes breast cancer cell migration, invasion, and stemness via increased TGF‐β1 secretion and subsequent TGF‐β/Smad pathway activation [183]. Conversely, high Cav‐1 expression in CAFs associates with poor prognosis in intrahepatic cholangiocarcinoma with increased infiltration of immunosuppressive Foxp3^+^ TILs [184], further illustrating context‐dependent duality.

### 4.3 Implications of CAF Functional Duality for Therapeutic Targeting

The recognition that CAFs encompass both tumor‐promoting and tumor‐restraining subsets has profound implications for therapeutic development [72]. Non‐selective targeting of CAFs, and the resulting abrupt degradation of ECM, can disrupt physical barriers that otherwise constrain tumor cells, thereby promoting invasion and metastasis [185, 186]. This is exemplified in PDAC models, where depletion of α‐SMA+ CAFs leads to more aggressive tumor phenotypes [177]. Thus, indiscriminate elimination of potentially beneficial CAF subsets disrupts microenvironmental homeostasis and may ultimately unleash more aggressive tumor behavior.

While ECM stiffness generally promotes tumor progression, select studies paradoxically report that reduced ECM components can accelerate malignancy [97, 103]. This duality may arise from compensatory protumorigenic signaling triggered by specific ECM alterations; for instance, Hedgehog pathway inhibition reduces PDAC stromal density but accelerates progression via enhanced angiogenesis [61, 187]. Similarly, while certain CAF subpopulations exhibit immunosuppressive properties, emerging evidence indicates that others may support antigen presentation or secrete chemokines that recruit immunostimulatory cells [178, 188]. These observations underscore the critical need for therapeutic strategies that selectively target pathogenic CAF subsets while preserving or even enhancing protective populations. Given the central role of CAF‐derived EVs in mediating many of these protumorigenic function, from metabolic reprogramming to immune suppression and therapy resistance, targeting the CAF‐EV communication axis represents a promising yet challenging therapeutic frontier requiring deeper investigation.

## 5 Therapeutic Strategies Targeting CAFs

**FIGURE 4:**
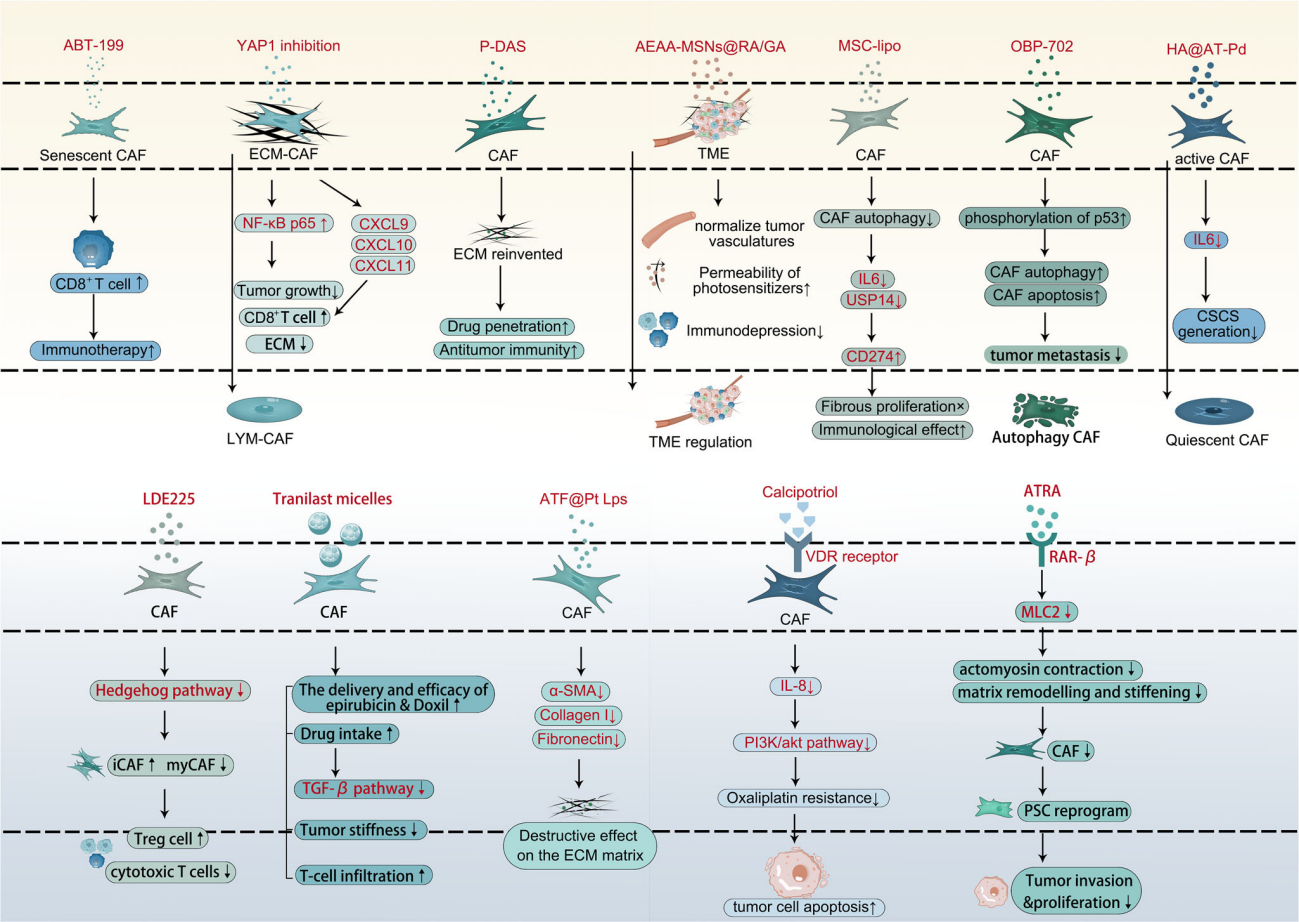
Emerging therapeutic strategies targeting cancer‐associated fibroblasts (CAFs) and stromal modulation. Diverse pharmacological and nanomedicine‐based approaches are developed to target CAF subtypes, remodel the extracellular matrix (ECM), and disrupt tumor‐stroma cross‐talk. CAF depletion and reprogramming: The Bcl‐2 inhibitor ABT‐199 targets senescent CAFs to enhance CD8^+^ T‐cell infiltration and immunotherapy efficacy. YAP1 inhibition activates NF‐κB p65 to upregulate CXCL9/10/11, promoting the recruitment of CD8^+^ T cells and inhibiting tumor growth. ECM reconstruction and vascular normalization: P‐DAS nanomedicines and ATF@Pt Lps (cisplatin liposomes) degrade the ECM matrix by downregulating α‐SMA, Collagen I, and Fibronectin, thereby improving drug penetration and antitumor immunity. AEAA‐MSNs@RA/GA normalize tumor vasculature to enhance photosensitizer permeability and alleviate immunodepression. Tranilast micelles diminish tumor stiffness and the TGF‐β pathway, facilitating T‐cell infiltration and drug delivery. Signaling and metabolic intervention: MSC‐lipo inhibits CAF autophagy to reduce IL‐6 and USP14 while upregulating CD274 (PD‐L1), enhancing immunological effects. OBP‐702 induces p53 phosphorylation to trigger CAF apoptosis and suppress metastasis. HA@AT‐Pd reduces IL‐6 secretion to inhibit cancer stem cell (CSC) generation and promote a transition to a quiescent CAF state. Conversely, the Hedgehog inhibitor LDE225 reduces myofibroblastic CAFs (myCAFs) but expands inflammatory CAF (iCAFs) and Tregs, leading to suppressed cytotoxic T‐cell function. Phenotypic normalization: Calcipotriol activates the vitamin D receptor (VDR) receptor to downregulate IL‐8 and the PI3K/Akt pathway, reversing oxaliplatin resistance and promoting tumor cell apoptosis. ATRA targets retinoic acid receptor‐beta (RAR‐β) to suppress myosin light chain 2 (MLC2), inhibiting actomyosin contraction and matrix remodeling to restrict tumor invasion and proliferation.

CAFs have emerged as compelling therapeutic targets due to their central role in orchestrating tumor progression, immune evasion, and therapy resistance. Studies using preclinical models have demonstrated promising anti‐tumor effects of CAF‐targeted treatments, leading to clinical investigations. However, their phenotypic plasticity, functional heterogeneity, and symbiotic relationship with tumor cells pose significant challenges for clinical therapeutic intervention. The current therapeutic landscape encompasses four broad strategies: direct depletion of CAF subsets, targeting CAF‐derived ECM components, disrupting CAF‐tumor cross‐talk, and inducing phenotypic normalization of CAFs (Table 2, Figure 4).

**TABLE 2:** Clinical landscape of CAF targeted therapeutic strategies.

Therapeutic strategy	Target/Pathway	Representative agents/interventions	Mechanism of action	Preclinical/Clinical evidence	Key challenges/Future directions
Direct CAF depletion	FAP	Anti‐FAP antibody (F19, sibrotuzumab)	Direct binding to FAP+ CAFs, antibody‐dependent cellular cytotoxicity (ADCC)	Phase II trials in advanced CRC showed no efficacy due to anti‐idiotypic antibodies^15^	Target selectivity limited (FAP also expressed on some immune cells); combination with immunotherapy warranted^189^, ^190^, ^191^
	FAP	177Lu‐LNC1004 (Evans blue‐modified FAPI)	Radioligand therapy targeting FAP+ cells	Phase I trial (NCT05963386) in metastatic thyroid cancer: ORR 25%, DCR 83%; scRNA‐seq showed enhanced antigen presentation but also T‐cell exhaustion^192^	Optimize dosing to balance efficacy and immune exhaustion; combine with ICIs
	FAP	FAP‐based DNA/DC vaccines	Break immune tolerance, promote CD4+/CD8+ T‐cell responses against CAFs	Preclinical: reduced tumor growth, enhanced T‐cell infiltration^190^, ^229^	Need for clinical translation; potential autoimmune toxicity
	FAP	FAP‐CAR T cells	Chimeric antigen receptor T cells targeting FAP+ CAFs	Preclinical: disrupted tumor stroma, enhanced chemotherapeutic uptake^190^, ^229^	Risk of on‐target off‐tumor toxicity (FAP expression in bone marrow); optimize CAR design
	Senescent CAFs	ABT‐199 (venetoclax, Bcl‐2 inhibitor)	Senolytic agent eliminating senescent CAFs	Preclinical: increased activated CD8+ T cells, reduced tumor burden in pancreatic cancer models; synergy with ICIs^230^	Identify optimal timing and patient selection; potential toxicity in normal tissues
ECM targeting	Hyaluronan (HA)	PEGPH20 (PEGylated recombinant human hyaluronidase)	Degrades HA to reduce interstitial pressure, improve vascular patency	Phase III trials in PDAC failed to show benefit with chemotherapy; combination with atezolizumab showed limited activity^201^	HA‐high tumors may benefit; need predictive biomarkers
	Tenascin‐C	131I‐m81C6 (anti‐tenascin monoclonal antibody)	Radiolabeled antibody targeting tenascin‐C in ECM	Phase II trial in recurrent glioma: improved median survival vs historical controls; no Phase III data reported	Limited to locoregional delivery; need more potent agents^197^
	Collagen/ECM	YAP1 inhibitors	Reprogram ECM‐depositing CAFs into immunostimulatory subtypes	Preclinical: reduced collagen density, enhanced CD8+ T‐cell infiltration	Specificity of YAP1 inhibition; potential developmental toxicity^194^
	Collagen/ECM	P‐DAS (dendritic polymer‐based nanomedicines)	Downregulate CAF‐derived collagen anabolism	Preclinical: improved drug penetration, amplified antitumor immunity	Nanomedicine delivery optimization
	Vascular normalization	AEAA‐MSNs@RA/GA	Targeted nanodrugs reducing collagen deposition and normalizing vasculature	Preclinical: enhanced photosensitizer accumulation, alleviated hypoxia, amplified photodynamic therapy	Translation to clinical settings; combination with other modalities^202^
	Vascular normalization	Losartan (angiotensin II receptor antagonist)	Inhibits CAF activation, reduces collagen, improves vascular permeability	Phase II study in locally advanced PDAC: 69.4% surgical resection rate, 61% R0 resection when added to FOLFIRINOX and chemoradiation^205^	Prospective validation needed; optimal combination regimen
Disrupting CAF‐tumor cross‐talk	TGF‐β	Bintrafusp alfa (M7824; bifunctional TGF‐β/PD‐L1 fusion protein)	Simultaneously blocks TGF‐β and PD‐L1	Promising Phase I data (40.7% ORR in PD‐L1+ NSCLC); subsequent Phase II/III trials failed to meet endpoints^16^, ^17^, ^207^, ^208^, ^209^	Patient selection crucial; compensatory pathways may limit efficacy
	TGF‐β	SHR1701 (PD‐L1/TGF‐β bispecific antibody)	Blocks both PD‐L1 and TGF‐β signaling	Phase III trial in NSCLC: 58.0% ORR with neoadjuvant SHR‐1701 plus chemotherapy in unresectable stage III disease^231^	Ongoing trials; biomarker development needed
	IL‐1	Anakinra (IL‐1 receptor antagonist)	Blocks IL‐1 signaling, reduces iCAF activation	Preclinical: increased CD8+ T cells within irradiated tumors; Phase I study in rectal cancer patients combining with chemoradiotherapy (ongoing)^212^	Optimal timing and combination with ICIs
	Hedgehog	Vismodegib, Sonidegib (LDE225)	Smoothened (SMO) inhibitors, block Hedgehog signaling	Approved for basal cell carcinoma; Preclinical: alter CAF subset composition (increase iCAFs, reduce CTL aggregation)^213^	Context‐dependent effects; may promote immunosuppression in certain settings
	Autophagy	MSC‐lipo (chloroquine liposomes targeting CAFs)	Inhibits CAF autophagy, reduces IL‐6 secretion	Preclinical: enhanced PD‐L1 expression, improved immunochemotherapy efficacy	Targeted delivery to CAFs; clinical translation pending^163^
	p53	OBP‐702 (oncolytic adenovirus)	Restores p53 function in CAFs, induces apoptosis	Preclinical: suppressed protumor cytokine secretion, reduced chemoresistance	Viral delivery; potential immunogenicity
	Metabolic reprogramming	HA@AT‐Pd nanomissile	Induces energy deprivation in cancer cells by suppressing OXPHOS and glycolysis	Preclinical: reduced lactate production, reversed immunosuppression	Nanomedicine optimization; combination potential^147^
	Metabolic reprogramming	CB‐839 (GLS1 inhibitor)	Glutamine starvation, impairs CAF‐mediated metabolic cross‐talk	Preclinical: enhances macropinocytosis‐mediated drug delivery	Biomarker development; combination strategies^147^
	CXCL12‐CXCR4	AMD3100 (plerixafor)	CXCR4 antagonist, blocks CAF‐mediated immune exclusion^158^	Preclinical: enhances T‐cell infiltration, synergizes with ICIs	Clinical trials in combination with ICIs ongoing
	IL‐6	Tocilizumab (anti‐IL‐6R antibody)	Blocks IL‐6 signaling, reduces CAF‐mediated immunosuppression	Preclinical: reduces M2 polarization, NETosis; clinical trials in cancer ongoing^163^, ^169^	Patient selection; combination with ICIs
Phenotype alteration	Vitamin D receptor (VDR)	Calcipotriol, Paricalcitol (VDR agonists)^215^, ^218^	Induce quiescent CAF phenotype, reduce αSMA expression, lipid droplet accumulation	Preclinical: enhanced gemcitabine efficacy in PDAC models; Pilot study: improved tumor vascularity and treatment efficacy	Ongoing clinical trials (NCT03300921, NCT03472833, NCT03520790, NCT02754726); biomarker development
	Retinoic acid receptor‐β (RAR‐β)	ATRA (all‐trans retinoic acid)^216^	Inhibits mechanosensory activation of stellate cells, reduces MLC2 contractility	Preclinical: reduced PSC activation, suppressed tumor growth in KPC model; Phase I: safe with chemotherapy, reduced neurotoxicity	Phase II trials ongoing (NCT04241276, NCT03572387)
	Tranilast	Tranilast‐loaded micelles^61^	Diminishes tumor stiffness, amplifies T‐cell recruitment	Preclinical: durable remission in resistant breast cancer models	Clinical translation; combination with ICIs
Diagnostic applications	FAP	68Ga‐FAPI PET/CT	Imaging agent for FAP+ stromal cells	Superior to 18F‐FDG in hepatic, gastric, pancreatic, and peritoneal cancers^190^, ^191^	Real‐time monitoring of CAF‐targeted therapies; guide treatment sequencing
	ECM stiffness	Elastography/MR imaging	Measure tumor stiffness as surrogate for CAF activity^103^, ^104^	Correlates with prognosis in CRC and other cancers	Standardization needed; prospective validation

Abbreviations: ATRA: all‐trans retinoic acid; CAR: chimeric antigen receptor; CRC: colorectal cancer; DCR: disease control rate; ECM: extracellular matrix; FAP: fibroblast activation protein; FAPI: FAP inhibitor; GLS1: glutaminase 1; HA: hyaluronan; HA: hyaluronic acid; ICI: immune checkpoint inhibitor; IL‐1/6: interleukin‐1/6; MLC2: myosin light chain 2; NSCLC: non‐small cell lung cancer; ORR: objective response rate; OXPHOS: oxidative phosphorylation; PDAC: pancreatic ductal adenocarcinoma; PD‐L1: programmed death‐ligand 1; PET/CT: positron emission tomography/computed tomography; PSC: pancreatic stellate cell; RAR‐β: retinoic acid receptor‐β; TGF‐β: transforming growth factor‐beta; VDR: vitamin D receptor.

### 5.1 Direct CAF Depletion Strategies

The most direct approach involves targeting CAF‐specific surface markers, with FAP emerging as a prominent candidate due to its selective expression on activated CAFs in most solid tumors [15, 189]. However, clinical translation has faced substantial hurdles. Several phase II trials in advanced colorectal cancer patients evaluating the anti‐FAP antibody sibrotuzumab failed to demonstrate efficacy, likely attributable to the development of anti‐idiotypic antibodies in treated patients [190, 191].

Recent innovations have focused on improving pharmacokinetic properties and therapeutic efficacy. The albumin‐binding Evans blue‐modified FAP inhibitor LNC1004, radiolabeled with 177Lu, was evaluated in a phase I trial (NCT05963386) for metastatic radioiodine‐refractory thyroid cancer, achieving objective response and disease control rates of 25% and 83%, respectively [15, 192]. Single‐cell RNA sequencing of post‐treatment samples revealed enhanced antigen processing and presentation but also T‐cell exhaustion, underscoring the need for combination approaches to overcome immune evasion [192].

Alternative FAP‐targeted strategies under investigation include FAP‐based DNA vaccines that break immune tolerance and promote CAF‐directed cytotoxic T‐cell responses, FAP‐directed dendritic cell vaccines that reduce TGF‐β signaling and enhance T‐cell activity, and FAP‐specific chimeric antigen receptor (CAR) T‐cell therapy that disrupts tumor stroma and improves chemotherapeutic uptake [190]. Beyond therapeutic applications, FAP inhibitors (FAPIs) have demonstrated diagnostic utility in PET/CT imaging, showing superiority to ^1^ ^8^F‐FDG for hepatic, gastric, pancreatic, and peritoneal cancers [15, 190, 191, 192].

Another emerging approach targets senescent CAFs, which accumulate in tumors and contribute to chemoresistance through senescence‐associated secretory phenotype (SASP) factors [65, 193]. Genetic or pharmacological depletion of senescent CAFs using the Bcl‐2 inhibitor ABT‐199 (venetoclax) increased activated CD8^+^ T‐cell proportions in mouse pancreatic carcinomas, and combining ABT‐199 with immune checkpoint blockade significantly reduced tumor burden [193]. These findings suggest that senolytic treatments may enhance immunotherapy efficacy.

### 5.2 Targeting CAF‐Derived ECM Components

CAFs are key producers of ECM components that create physical barriers to drug delivery and immune cell infiltration. Hyaluronan (HA) and tenascin C (TNC) represent major targets for therapeutic intervention YAP1 inhibition reprograms ECM‐depositing CAFs toward immunostimulatory phenotypes, reducing collagen density and enhancing CD8^+^ T‐cell infiltration [194]. Dendritic polymer‐based nanomedicines (P‐DAS) downregulate CAF‐derived collagen anabolism, improving drug penetration and amplifying antitumor immunity [195]. Human recombinant PH20 hyaluronidase (PEGPH20) depletes HA in combination with nab‐paclitaxel and gemcitabine, reducing vascular compression and improving immune cell and drug entry into tumor vasculature [196]. Tenascin‐C inhibition sensitizes T cell‐mediated tumor killing and improves anti‐PD‐1/PD‐L1 therapy efficacy [197]. In recurrent malignant glioma, administration of 131I‐m81C6 (iodine‐131‐labeled murine anti‐tenascin monoclonal antibody) followed by chemotherapy was associated with median survival exceeding historical controls in a single‐arm phase II tria [198].

Despite these promising preclinical and early clinical results, translation to practice has proven disappointing. No positive phase III results for 131I‐m81C6 have been reported, and two separate clinical trials failed to demonstrate significant benefit of PEGPH20 combined with standard‐of‐care chemotherapy in advanced PDAC [199, 200]. Similarly, the combination of atezolizumab and PEGPH20 showed limited clinical activity in PDAC and no efficacy in gastric cancer [201].

Vascular normalization represents a complementary strategy to counteract CAF‐driven progression by restoring vascular integrity and stromal balance. Nanodrugs (AEAA‐MSNs@RA/GA) targeting CAFs reduce collagen deposition and normalize tumor vasculature, enhancing photosensitizer accumulation and alleviating hypoxia to amplify photodynamic therapy efficacy [202]. Losartan‐mediated CAF inhibition combined with CO release improved vascular permeability and nanoparticle penetration in solid tumors [203]. Adding losartan to FOLFIRINOX and chemoradiation was associated with high rates of surgical resection and R0 resection in locally advanced PDAC [204].

### 5.3 Disrupting CAF‐Tumor Cross‐Talk

The reciprocal interaction between CAFs and tumor cells sustains a self‐reinforcing network that drives malignant progression through multiple signaling pathways TGF‐β signaling is central to CAF activation, prompting development of TGF‐β inhibitors [205]. Blocking TGF‐β also shows potential to augment ICI responses due to its well‐documented immunosuppressive effects [206]. Bintrafusp alfa, a bifunctional fusion protein inhibiting both TGF‐β and PD‐L1, showed promising phase I results (40.7% objective response rate in PD‐L1‐positive NSCLC) but subsequently failed to meet clinical thresholds in several phase II and III trials [206, 207, 208]. However, other PD‐L1/TGF‐β bispecific antibodies such as SHR1701 continue to show promise, achieving 58.0% ORR when combined with neoadjuvant chemotherapy in unresectable stage III NSCLC [209]. These mixed outcomes suggest that patient selection and mechanism‐driven study design are critical for future TGF‐β‐targeted approaches [205].

Autophagy inhibition in CAFs using chloroquine diphosphate‐loaded liposomes (MSC‐lipo) reduces IL‐6 secretion, enhancing PD‐L1 expression and immunochemotherapy efficacy [210]. p53 restoration in CAFs through oncolytic adenoviruses (OBP‐702) suppresses protumorigenic cytokine secretion and chemoresistance [167]. Metabolic intervention represents a promising strategy to interrupt CAF‐tumor metabolic cross‐talk. The nanomissile HA@AT‐Pd induces energy deprivation in breast cancer cells by suppressing oxidative phosphorylation and glycolysis, reducing lactate production and reversing immunosuppression [147]. Glutamine starvation via the GLS1 inhibitor CB‐839 enhances macropinocytosis‐mediated drug delivery while impairing CAF‐mediated metabolic support [147].

Immune modulation approaches targeting CAFs aim to reverse immunosuppressive TME and enhance antitumor immunity. IL‐1 receptor antagonist anakinra increased CD8^+^ T cells within irradiated tumors, and its combination with chemoradiotherapy is under investigation in rectal cancer (phase I) [211]. Hedgehog inhibitors vismodegib and sonidegib, approved for basal cell carcinoma, alter CAF subset composition, increasing iCAFs while attenuating cytotoxic T lymphocyte aggregation [212, 213]. Tranilast‐loaded micelles diminish tumor stiffness and amplify T‐cell recruitment, achieving durable remission in resistant breast cancer models [61]. Cisplatin liposomes degrade stromal barriers to enhance CD8^+^ T‐cell infiltration and anti‐PD‐1 response in pancreatic cancer [211]. These strategies collectively convert “cold” tumors into immunogenic “hot” landscapes, overcoming CAF‐mediated therapeutic resistance [214].

### 5.4 Phenotypic Normalization of CAFs

Transforming activated CAFs into quiescent states represents an emerging therapeutic paradigm that preserves stromal integrity while eliminating protumorigenic functions [215, 216]. Vitamin D receptor (VDR) agonists induce CAF quiescence characterized by lipid droplet accumulation and reduced α‐SMA expression Sherman et al. demonstrated that CAFs in human PDAC express high VDR levels, and treatment with calcipotriol (VDR agonist) induced quiescent phenotypes [215]. Paricalcitol, a synthetic vitamin D analogue, significantly enhanced gemcitabine efficacy in xenograft and orthotopic PDAC models by increasing intratumoral concentrations of active metabolite dFdCTP [215]. A pilot study reported that higher paricalcitol doses (7 mcg/kg/week) combined with Nal‐IRI and 5‐FU/LV were well‐tolerated, improved tumor vascularity, and potentially enhanced treatment efficacy in PDAC [217]. Beyond pancreatic cancer, VDR activation in gastric cancer reversed CAF‐derived IL‐8‐mediated oxaliplatin resistance by inhibiting PI3K/Akt signaling [218]. Ongoing clinical trials are evaluating vitamin D supplementation in resectable (NCT03300921, NCT03472833) and unresectable PDAC (NCT03520790, NCT02754726).

All‐trans retinoic acid (ATRA) inhibits mechanosensory activation of pancreatic stellate cells and reduces stromal remodeling capacity through RAR‐β‐mediated downregulation of MLC2 contractility [217]. In vivo, ATRA reduced PSC activation and motility, disrupted Wnt signaling in cancer cells, and suppressed tumor growth in KPC PDAC models [216]. A phase I study demonstrated ATRA's safety and tolerability in combination with gemcitabine‐nab‐paclitaxel, with enhanced chemotherapy dose intensity and reduced neurotoxicity [219]. ATRA efficacy is being further evaluated in phase II trials for locally advanced PDAC (NCT04241276) and prostate cancer with PSA‐only recurrence (NCT03572387).

### 5.5 Clinical Challenges and Future Directions

Developing effective CAF‐targeted therapies presents multiple clinical challenges [190, 203, 219]. First, identifying highly selective targets remains difficult, as markers such as FAP and CD10 are also expressed on certain cancer cells and immune cells, potentially leading to off‐target effects and misclassification. Second, CAF subset heterogeneity complicates therapeutic precision; for example, chemoresistance‐inducing CD10^+^GPR77^+^ CAFs are activated via TAM‐secreted CCL18, requiring subset‐specific targeting strategies [220, 221]. Third, CAF cross‐talk with immune cells and dynamic TME remodeling necessitates real‐time monitoring strategies, such as 68Ga‐FAPI PET/CT‐guided interventions, to optimize treatment sequencing while minimizing toxicity [222].

Compensatory mechanisms have contributed to numerous clinical trial failures. Col6a1^+^ CAFs maintain homeostatic features while acquiring proangiogenic functions, and TNFR1/IL‐1R inhibition fails to block tumorigenesis due to compensatory activation of other subsets [223]. Adenosine signaling blockade in melanoma triggers compensatory CD39/CD73 upregulation, sustaining protumorigenic adenosine levels [224]. In lung cancer, CAF‐secreted IGFBPs initially sensitize tumors, but residual IGF1R/FAK signaling drives resistance, highlighting context‐dependent compensatory cross‐talk [225]. These adaptive responses underscore the need for multitargeted approaches combining CAF‐directed therapies with ICIs, chemotherapy, or other stromal modulators [72].

## 6 Future Perspectives

The future of CAF‐targeted therapies lies in integrated approaches that leverage technological advances and biological insights to overcome heterogeneity and adaptive resistance.

Single‐cell multiomics integration will enable comprehensive mapping of CAF heterogeneity across malignancies. By coupling scRNA‐seq, proteomics, and spatial transcriptomics, researchers can delineate subtype‐specific signatures, identify novel druggable targets, and track dynamic phenotypic shifts during therapy [72]. Multiomic analyses will unravel context‐dependent CAF‐tumor‐immune cross‐talk, including metabolic symbiosis (lactate/glutamine shuttling) and niche‐specific signaling hubs [132] Machine learning models trained on multiomic datasets could predict compensatory pathway activation and guide rational combinatorial regimens. Clinically, this approach enables patient stratification based on CAF subtype composition, moving toward precision stroma‐targeted therapy [192].

Spatial biology applications will elucidate the architectural organization of CAF‐tumor‐immune ecosystems. Spatial transcriptomics and multiplexed imaging can map CAF subtypes (myCAFs, iCAFs, and apCAFs) within distinct stromal niches, revealing location‐dependent functions, such as perivascular CAFs driving angiogenesis or invasive‐front CAFs promoting EMT [70]. These tools decode ligand‐receptor networks at cellular‐resolution interfaces, identifying spatially constrained therapeutic vulnerabilities. Spatial metabolomics may uncover niche‐specific metabolic cross‐talk, while AI‐driven spatial modeling predicts how stromal perturbations alter biomechanical signaling (YAP/TAZ) or immune exclusion zones. Ultimately, spatial mapping could guide precise delivery of CAF‐directed agents and suggest rational combination treatments by linking CAF locations to resistance patterns [214].

Patient‐derived models will become increasingly central to CAF‐targeted drug development. Organoids, patient‐derived xenografts (PDX), and 3D co‐cultures preserve native ECM architecture and metabolic symbiosis, enabling more physiologically relevant testing of drug responses [219, 226]. Combining patient‐derived models with single‐cell and spatial omics can reveal how therapy alters CAF behavior and identify resistance mechanisms such as compensatory Wnt or YAP activation. Personalized models also facilitate biomarker discovery by linking CAF secretomes (IL‐6, exosomal miRNAs) to patient‐specific outcomes However, challenges remain in scaling these systems for high‐throughput drug screening.

Combination therapy optimization will be essential to overcome both inherent CAF heterogeneity and adaptive resistance mechanism. Dual inhibition of complementary pathways may block compensatory signaling, while pairing CAF modulators with ICIs (anti‐PD‐1/CTLA‐4) could synergize to reverse stromal immunosuppression [119, 214]. Preclinical models demonstrate efficacy in combining ECM‐disrupting agents (LOX inhibitors) with chemotherapy to enhance drug penetration [227] or pairing metabolic interventions (MCT4 blockade) with anti‐angiogenics to starve tumors [228]. AI‐driven platforms analyzing multiomics datasets will predict optimal drug combinations by mapping CAF‐tumor‐immune cross‐talk networks and forecasting resistance mechanisms.

Taken together, the convergence of single‐cell technologies, spatial biology, patient‐derived models, and computational approaches will transform CAF targeting from a one‐size‐fits‐all strategy into context‐driven precision therapy. Modular trial designs incorporating real‐time stromal monitoring via liquid biopsies may refine dynamic combination approaches, ultimately realizing the therapeutic potential of targeting the tumor stroma while avoiding the pitfalls of non‐selective CAF depletion [177].

Figure production: Images were generated using Adobe Photoshop software (version [CC 2019]; Adobe Inc., San Jose, CA, USA).

## Funding

This work was supported by the National Key R&D Program of China (grant number 2023YFC2506400), National Natural Science Funds (grant number 82225038, 82430092, 82272828, 82172735 and M‐0349), Shanghai Science and Technology Innovation Action Plan (grant number 23J21900900), National Science Fund for Distinguished Young Scholars Fund (grant number 82125026), Innovative research team of high‐level local universities in Shanghai (grant number SHSMU‐ZLCX20211600), Talent Introduction Fund of Fudan University Shanghai Cancer Center (grant number YJRC202403) and Shanghai Anticancer Association SOAR PROJECT (SACA‐AX202401).

## Conflicts of Interest

The authors declare no conflicts of interest.

## Data Availability

The authors have nothing to report.
